# Exogenous Hydrogen Sulfide Within the Nucleus Ambiguus Inhibits Gastrointestinal Motility in Rats

**DOI:** 10.3389/fphys.2020.545184

**Published:** 2020-09-11

**Authors:** Hongzhao Sun, Haikun Ding, Yuan Shi, Chenyu Li, Haoran Jin, Xiaoyue Yang, Zhaosong Chen, Pengpeng Tian, Jianping Zhu, Haiji Sun

**Affiliations:** ^1^School of Life Sciences, Qilu Normal University, Jinan, China; ^2^Key Laboratory of Animal Resistance, School of Life Sciences, Shandong Normal University, Jinan, China

**Keywords:** nucleus ambiguus, hydrogen sulfide, gastric motility, TRPV1, NF-κB

## Abstract

Hydrogen sulfide (H_2_S) is a neuromodulator in the central nervous system. However, the physiological role of H_2_S in the nucleus ambiguus (NA) has rarely been reported. This research aimed to elucidate the role of H_2_S in the regulation of gastrointestinal motility in rats. Male Wistar rats were randomly assigned to sodium hydrosulfide (NaHS; 4 and 8 nmol) groups, physiological saline (PS) group, capsazepine (10 pmol) + NaHS (4 nmol) group, L703606 (4 nmol) + NaHS (4 nmol) group, and pyrrolidine dithiocarbamate (PDTC, 4 nmol) + NaHS (4 nmol) group. Gastrointestinal motility curves before and after the injection were recorded using a latex balloon attached with a pressure transducer, which was introduced into the pylorus through gastric fundus. The results demonstrated that NaHS (4 and 8 nmol), an exogenous H_2_S donor, remarkably suppressed gastrointestinal motility in the NA of rats (*P* < 0.01). The suppressive effect of NaHS on gastrointestinal motility could be prevented by capsazepine, a transient receptor potential vanilloid 1 (TRPV1) antagonist, and PDTC, a NF-κB inhibitor. However, the same amount of PS did not induce significant changes in gastrointestinal motility (*P* > 0.05). Our findings indicate that NaHS within the NA can remarkably suppress gastrointestinal motility in rats, possibly through TRPV1 channels and NF-κB-dependent mechanism.

## Introduction

Hydrogen sulfide (H_2_S) is an essential neuromodulator in mammals (Zheng et al., 2018), which regulates numerous pathophysiological functions in the digestive, respiratory, circulatory and nervous systems (Kimura et al., 2012; Drapala et al., 2017; Yang et al., 2017; Yu et al., 2017; Zhou et al., 2018). Endogenous H_2_S levels have been detected in the brains of rats and humans (Wang, 2012; Yang and He, 2019). The concentrations of endogenous H_2_S are typically 47–166 μmol L^–1^ in the brain (Furne et al., 2008). Free H_2_S is ≤ 9.2 μM in the brain. The release of H_2_S was maximal at pH 1.5 and gradually decreased with higher pH up to 5.4. H_2_S released by HCl alone was 161 ± 5 nmol/g protein, whereas that by DTT alone was 1,481 ± 174 nmol/g protein (Warenycia et al., 1989; Ishigami et al., 2009). And such high content suggests that H_2_S may exert a physiological function (Goodwin et al., 1989; Fiorucci et al., 2006).

H_2_S is a gasotransmitter produced primarily by cystathionine-β-synthase (CBS) enzyme in the CNS (Swaroop et al., 1992; Calvert et al., 2010; Wang, 2012). Endogenous H_2_S affects cardiac function in the nucleus tractus solitarii by regulating ATP-sensitive potassium (K_*ATP*_) channels and/or glutamate receptors (Qiao et al., 2011). Microinjecting L-glutamate into the nucleus ambiguus (NA) can suppress gastrointestinal motility via activating specific N-methyl-D-aspartate receptor (Sun et al., 2010). In our previous works (please see Supplementary Files), we found that CBS is localized in NA. These experimental findings indicate that H_2_S in the NA can participate in the regulation of gastric functions.

H_2_S regulates many physiological and pathological processes through ion channels. Several recent publications indicated the ability of H_2_S to activate TRPV1 or TRPA1 receptors *in vitro* and *in vivo* experiments (Koroleva et al., 2017). Thus, the TRPV1 antagonist prevented NaHS-evoked luminal chloride secretion (Storti et al., 2015). It initiates the activation of transient receptor potential vanilloid 1 (TRPV1) channels in the urinary bladder of rats and airway of guinea pigs, thus leading to bladder obstruction and airway constriction via neurogenic inflammation (Patacchini et al., 2004; Trevisani et al., 2005). NaHS promotes gastric acid secretion by activating TRPV1 channels in the sensory nerve endings with the subsequent release of substance P (SP) in an NF-κB-dependent manner (Sun et al., 2018). Lu et al. (2014) found that capsazepine, TRPV1 channel antagonists, and L703606, a NK1 receptor antagonist, significantly attenuated the excitatory responses evoked by NaHS, which indicates that NaHS might activate TRPV1 channels in the afferent nerve fibers with the consequent release of SP. However, the mechanism underlies H_2_S-induced regulation of gastric motility in the NA remains largely unclear.

The transcription factor NF-κB is a pleiotropic mediator of target genes that modulates many physiological functions (Gutierrez et al., 2005; Meffert and Baltimore, 2005; Blank and Prinz, 2014; Haenold et al., 2014). H_2_S protects gastric mucosal cells from ischemia-reperfusion damage by regulating NF-κB-dependent anti-inflammatory activity. Besides, Keap1 S-sulfhydration anti-apoptosis pathway also protects against water-immersion and restraint stress-induced gastric damage in rats, by opening K_*ATP*_ channel and activating NF-κB-dependent pathway (Guo et al., 2014). Ang et al. (2011) have reported that H_2_S can regulate TRPV1-induced neurogenic inflammation in sepsis by enhancing SP production and activating ERK-NF-κB pathway. This study aimed to elucidate the role of H_2_S in regulating gastrointestinal motility in rats, and to investigate whether the effect of H_2_S is resulted from the activation of TRPV1 channels though a NF-κB-dependent manner.

## Materials and Methods

### Subjects

Wistar rats (male; weighing 270–320 g) were supplied by the Experimental Animal Center of Shandong University. The animals were housed at a constant temperature under a 12:12 h light–dark cycle, and were given *ad libitum* access to water and food for 7 days. Before starting the experiments, the rats were deprived of food for 24 h. Ethical approval for this study was obtained from the Experimental Animal Ethics Committee of Qilu Normal University, and all experimental protocols were conducted in accordance with the guidelines of the International Association for the Study of Pain (Zimmermann, 1986).

### Chemicals

NaHS (4 and 8 nmol) (Sun et al., 2015), capsazepine (10 pmol), L703606 (4 nmol), PDTC (4 nmol), and pontamine sky blue were all supplied by Sigma-Aldrich (St. Louis, MO, United States). NaHS was dissolved in 0.9% saline, while other chemicals were dissolved in DMSO.

### Recording of Gastrointestinal Motility and Microinjection Studies

Anesthetic procedure was carried out by intraperitoneally injecting chloral hydrate (400 mg/kg body weight) into the rats. Laparotomy was performed, and a warm water-filled balloon (5 mm in diameter) was introduced into the pylorus via a small incision in the forestomach wall. Gastrointestinal motility curves were determined using BL-420F (Biological Function Experimental System; Chengdu Taimeng Company, China) through a pressure transducer.

The anesthetized rats were transferred into a stereotaxic apparatus (Stoelting 68002, Shenzhen Ruiwode Company, China). After performing an occipital craniotomy, a glass micropipette (external tip diameter: 30–50 μm) with pneumatic pump was placed vertically in the right NA. The position was identified based on the Paxinos and Watson rat brain atlas (Paxinos and Watson, 2007).

### Experimental Group

A series of experiments was perform to assess the effects of H_2_S on gastrointestinal motility in the NA. (i) Microinjection of NaHS (0.1 μL, 4 nmol) into the NA (*n* = 6); (ii) microinjection of NaHS (0.1 μL, 8 nmol) into the NA (*n* = 5); (iii) microinjection of physiological saline (PS; 0.1 μL) the NA (*n* = 4) as control group; (iv) microinjection of NaHS (0.1 μL, 4 nmol) and capsazepine (0.1 μL, 10 pmol) into the NA (*n* = 6); (v) microinjection of L703606 (0.1 μL, 4 nmol) and NaHS (0.1 μL, 4 nmol) into the NA (*n* = 5); and (vi) microinjection of PDTC (0.1 μL, 4 nmol) and NaHS (0.1 μL, 4 nmol) into the NA (*n* = 5).

### Histological Analysis of the Microinjection Site

After completion of each experiment, 2% pontamine sky blue (0.1 μL) was administered at the same microinjection site. The reaction was terminated though an intravenous bolus injection of 80 mg/kg pentobarbital sodium. Next, the rats were transcardially perfused with PS, followed by fixation in 4% paraformaldehyde. Then, the brains were isolated and placed in 4% paraformaldehyde containing 20% sucrose for approximately 48–72 h. The brainstem samples (40 μm in thickness) were snap-frozen and sectioned, followed by neutral red staining. Statistical analyses were performed for the results in which the tip of the microinjection site was localized within the NA.

### Data Analysis

The total amplitude and total duration of gastric contraction waves were measured 5 min pre- and post-microinjection. Gastric motility index (GMI) was calculated as a function of the amplitude and duration of each contraction wave. To determine the changes in gastrointestinal motility parameters pre- and post-microinjection, the rates of inhibition were calculated as follows: Inhibition rate (%) = (pre-microinjection value – post-microinjection value) × 100% / pre-microinjection value (Sun et al., 2015).

SPSS v23.0 (IBM SPSS Inc., Chicago, IL, United States) was employed to analyze the results. Statistical differences were evaluated using Student’s *t*-test followed by *post hoc* testing with the Student–Newman–Keuls test. All data were presented as mean ± standard error. *P* values of less than 0.05 was deemed as statistically significant.

## Results

### NaHS Inhibits Gastrointestinal Motility Within NA

**FIGURE 1:**
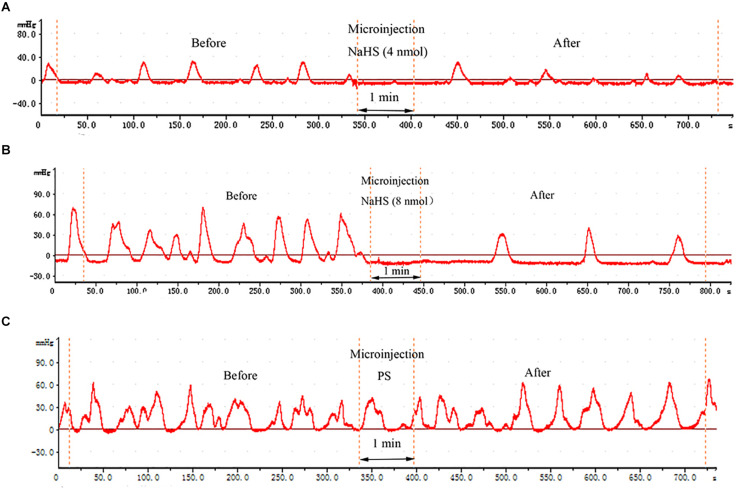
Effects of chemicals microinjected into the nucleus ambiguus (NA) on gastric motility. Effects of NaHS (4 nmol in 0.1 μL) microinjected into the NA on gastric motility (representing curve from a rat) **(A)**. Effects of NaHS (8 nmol in 0.1 μL) microinjected into the NA on gastric motility (representing curve from a rat) **(B)**. Representative curve from a rat showing the Effects of 0.1 μL physiological saline (PS) microinjected into the NA on gastric motility (representing curve from a rat) **(C)**.

As shown in Figures 1A,B, NaHS (4 and 8 nmol), administered at the right NA, was found to exert remarkable inhibitory effect on gastrointestinal motility. In contrast, the same amount of PS exhibited no significant effect on gastrointestinal motility (Figure 1C).

**FIGURE 2:**
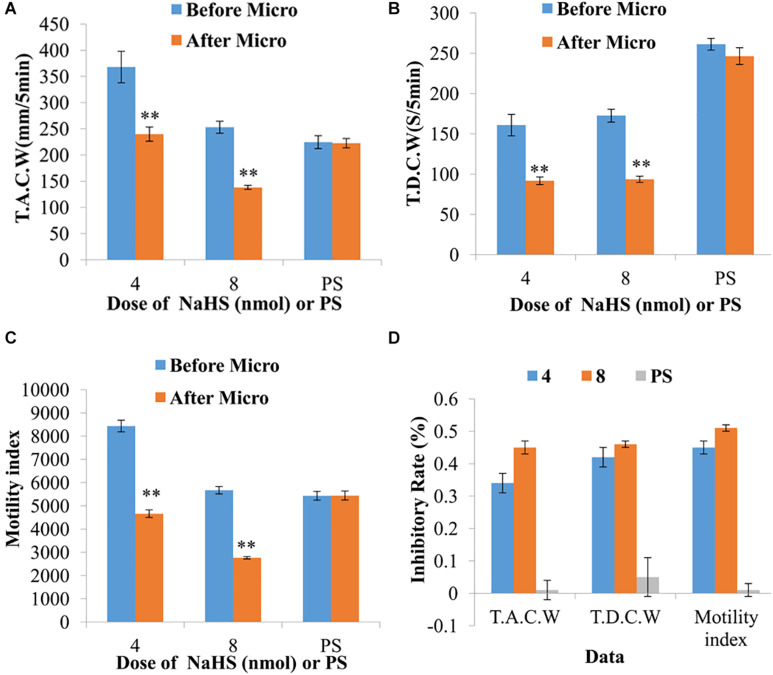
Representative data for gastric motility before and after microinjection of NaHS (4 and 8 nmol) or physiological saline (PS) into the nucleus ambiguus. Data of T.A.C.W **(A)**. Data for T.D.C.W **(B)**. Data for gastric motility index **(C)**. Average inhibitory rate of T.A.C.W, T.D.C.W and gastric motility index **(D)**. Micro: microinjection; T.A.C.W: total amplitude of contraction waves; T.D.C.W: total duration of contraction waves; ^∗∗^*P* < 0.01, versus before microinjection.

Moreover, the data measured 5 min pre- and post-microinjection were analyzed and compared. At a dose of 4 nmol NaHS, the total amplitude of contraction waves (TACW), total duration of contraction waves (TDCW) and GMI were declined from 367.89 ± 30.03 mm 5 min^–1^ to 239.87 ± 13.51 mm 5 min^–1^ (*P* < 0.01), 160.88 ± 13.33 s 5 min^–1^ to 91.68 ± 4.73 s 5 min^–1^ (*P* < 0.01) and 8435.57 ± 251.48 to 4662.21 ± 164.32 (*P* < 0.01), respectively, before and after microinjection (Figures 2A–C). At a dose of 8 nmol NaHS, the values of TACW, TDCW and GMI reduced from 253.04 ± 11.39 mm 5 min^–1^ to 138.20 ± 3.85 mm 5 min^–1^ (*P* < 0.01), 172.60 ± 7.99 s 5 min^–1^ to 93.60 ± 3.85 s 5 min^–1^ (*P* < 0.01), and 5670.14 ± 159.82 to 2766.05 ± 49.35 (*P* < 0.01), respectively, before and after microinjection (Figures 2A–C).

As displayed in Figure 2D, the inhibitory rates of TACW in 4 and 8 nmol NaHS groups were found to be 33.95 and 45.07%, respectively. The inhibitory rates of TDCW in 4 and 8 nmol NaHS groups were determined to be 42.03 and 45.68%, respectively. The inhibitory rates of GMI in 4 and 8 nmol NaHS groups were observed to be 44.54 and 51.14%, respectively. Notably, the inhibitory rates of TACW, TDCW, and GMI in 4 nmol NaHS group were relatively lower than those in 8 nmol NaHS group. These results indicate that NaHS within the NA may have a dose-dependent trend in gastrointestinal motility.

### Capsazepine Prevents the Suppressive Effect of NaHS on Gastrointestinal Motility

**FIGURE 3:**
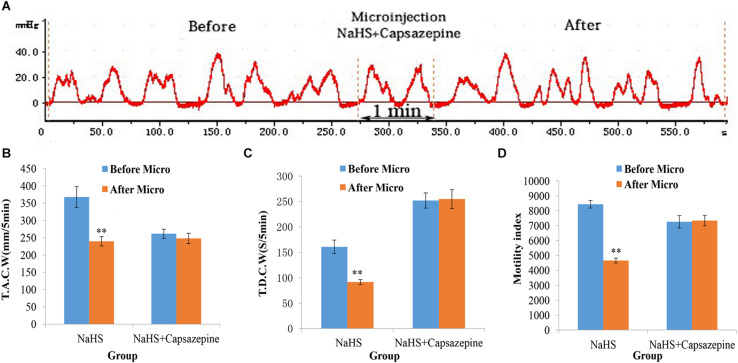
Representative data for gastric motility in the NaHS (4 nmol in 0.1 μL) group and NaHS (4 nmol in 0.1 μL) + Capsazepine group. Gastric motility curve in NaHS + Capsazepine group **(A)**. Data of T.A.C.W **(B)**. Data for T.D.C.W **(C)**. Data for gastric motility index **(D)**. Micro: microinjection; T.A.C.W: total amplitude of contraction waves; T.D.C.W: total duration of contraction waves; ^∗∗^*P* < 0.01, versus before microinjection.

Pretreatment with capsazepine (a TRPV1 antagonist) in the NA could attenuate the suppressive effect of NaHS on gastrointestinal motility (Figure 3A). As shown in Figures 3B–D, the values of TACW changed from 261.71 ± 13.09 mm 5 min^–1^ (pre-microinjection) to 248.01 ± 15.04 mm 5 min^–1^ (post-microinjection, *P* > 0.05); those of TDCW changed from 252.03 ± 14.89 s 5 min^–1^ (pre-microinjection) to 254.95 ± 18.56 s 5 min^–1^ (post-microinjection, *P* > 0.05); and those of GMI changed from 7262.51 ± 415.66 (pre-microinjection) to 7342.42 ± 346.59 (post-microinjection, *P* > 0.05). These findings reveal that NaHS can regulate gastrointestinal motility through TRPV1 channels.

### L703606 Dispels the Suppressive Effect of NaHS on Gastrointestinal Motility

**FIGURE 4:**
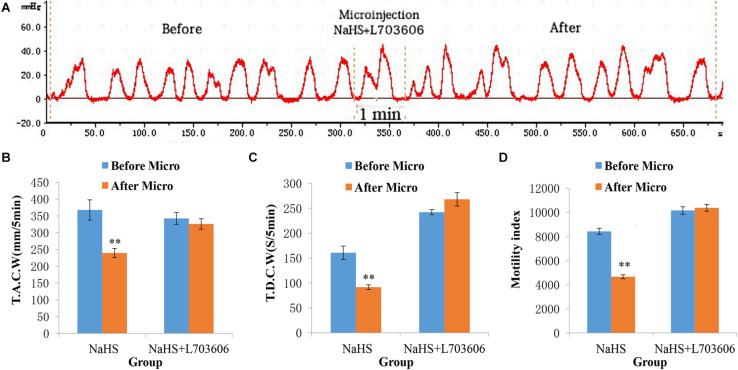
Representative data for gastric motility in the NaHS (4 nmol in 0.1 μL) group and NaHS (4 nmol in 0.1 μL) + L703606 group. Gastric motility curve in NaHS + L703606 group **(A)**. Data of T.A.C.W **(B)**. Data for T.D.C.W **(C)**. Data for gastric motility index **(D)**. Micro: microinjection; T.A.C.W: total amplitude of contraction waves; T.D.C.W: total duration of contraction waves; ^∗∗^*P* < 0.01, versus before microinjection.

The inhibitory effect of NaHS on gastrointestinal motility could also be reduced by pretreatment with L703606 (a NK1 receptor antagonist; Figure 4A). As shown in Figures 4B–D, The values of TACW changed from 342.51 ± 17.49 mm 5 min^–1^ (pre-microinjection) to 326.13 ± 15.72 mm 5 min^–1^ (post-microinjection, *P* > 0.05); those of TDCW changed from 242.20 ± 5.20 s 5 min^–1^ (pre-microinjection) to 268.00 ± 13.47 s 5 min^–1^ (post-microinjection, *P* > 0.05); and those of GMI changed from 10164.75 ± 310.12 (pre-microinjection) to 10385.65 ± 278.73 (post-microinjection, *P* > 0.05). These findings demonstrate that NaHS can regulate gastrointestinal motility by enhancing SP production.

### PDTC Eliminates the Suppressive Effect of NaHS on Gastrointestinal Motility

**FIGURE 5:**
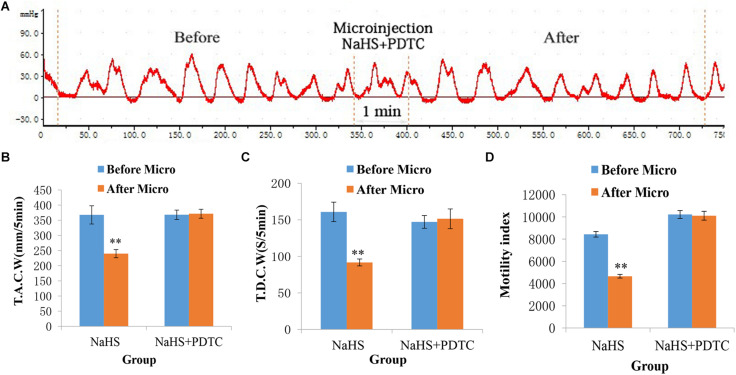
Representative data for gastric motility in the NaHS (4 nmol in 0.1 μL) group and NaHS (4 nmol in 0.1 μL) + PDTC group. Gastric motility curve in NaHS + PDTC group **(A)**. Data of T.A.C.W **(B)**. Data for T.D.C.W **(C)**. Data for gastric motility index **(D)**. Micro: microinjection; T.A.C.W: total amplitude of contraction waves; T.D.C.W: total duration of contraction waves; PDTC: pyrrolidine dithiocarbamate; ^∗∗^*P* < 0.01, versus before microinjection.

The gastrointestinal motility did not change significantly after administration of PDTC into the NA (Figure 5A). As shown in Figures 5B–D, the values of TACW changed from 368.29 ± 15.44 mm 5 min^–1^ (pre-microinjection) to 371.68 ± 14.81 mm 5 min^–1^ (post-microinjection, *P* > 0.05); those of TDCW changed from 147.20 ± 8.72 s 5 min^–1^ (pre-microinjection) to 151.40 ± 13.52 s 5 min^–1^ (post-microinjection, *P* > 0.05); and those of GMI changed from 10216.24 ± 354.10 (pre-microinjection) to 10107.41 ± 396.45 (post-microinjection, *P* > 0.05). These findings imply that NaHS may govern gastrointestinal motility by activating NF-κB signaling pathway.

## Discussion

This study provides strong evidence that exogenous H_2_S is involved in the regulation of gastrointestinal motility within the NA of rats. The values of TACW, TDCW, and GMI were significantly reduced following microinjection of 4 and 8 nmol NaHS (an exogenous H_2_S donor) into the NA. However, the frequency of gastrointestinal motility did not change regularly. Two doses (4 and 8 nmol) of NaHS were determined from the pretest values and literature data (Sun et al., 2015). The dose-dependent microinjection of NaHS into the NA remarkably suppressed gastrointestinal motility in rats. Our previous experiments have suggested that CBS, which responsible for H_2_S production, is highly expressed in the dorsal motor nucleus of the vagus (DMV), and the microinjection of NaHS into the DMV could inhibit gastrointestinal motility and promote gastric acid secretion in rats (Sun et al., 2018). Altogether, these findings indicate that H_2_S within the NA may act as a neuromodulator or transmitter to inhibit the regulation of gastrointestinal motility.

As the centrum of the parasympathetic preganglionic nerve, the NA and DMV, both act effectively on gastric motility (Zhang et al., 2006, 2009; Wang et al., 2007; Sun et al., 2009), which can represent a source of vagal innervation in the gut. The importance of the activity of this autonomic output in regulating gastrointestinal motility has been well-established (Sun et al., 2010). The vagus nerve is the main autonomic pathway through which these effects are eventually mediated by the NA. Both excitatory and inhibitory fibers are found in the vagus nerves (Cruz et al., 2007). It is speculated that the excitatory fibers may be inhibited, while the inhibitory fibers are excited in the vagus nerve following microinjection of NaHS into the NA.

The present findings revealed that capsazepine, a TRPV1 antagonist, could abolish the suppressive effect of NaHS on gastrointestinal motility, indicating that the suppressive effect of NaHS on gastrointestinal motility is regulated by the initiation of TRPV1 channels. TRPV1 channels are members of the ligand-gated ion channel family, which not only driven by the binding of specific lipophilic molecules but also serve as extracellular protons and physical stimuli, such as heat and osmotic stress (Kauer and Gibson, 2009). Most studies have highlighted on the structure and function of TRPV1, but other thermosensitive TRPV subunits, such as TRPV2, TRPV3 and TRPV4, are also found in the CNS (Caterina, 2007). Besides, activation of TRPV1 receptors can increase glutamate release in the DMV (Derbenev et al., 2006; Derbenev and Smith, 2013). Our previous study has found that microinjection of L-glutamate or NaHS into the DMV can inhibit gastrointestinal motility (Sun et al., 2015). These reports suggest that the microinjection of NaHS into the DMV may increase glutamate release by activating TRPV1 receptors, and the vagal parasympathetic nerve fibers innervating gastric smooth muscles are from both DMV and NA. Thus, it is feasible that the microinjection of NaHS into the NA can inhibit gastrointestinal motility via TRPV1 receptor activation.

Our results demonstrated that the suppressive effect of NaHS on gastrointestinal motility could be prevented by PDTC, an NF-κB inhibitor, suggesting that NaHS may act in an NF-κB-dependent manner. H_2_S, a gaseous transmitter, can diffuse freely across various biological membranes and regulate numerous signaling pathways, including NF-κB signaling pathway (Calvert et al., 2010; Bos et al., 2012). Typically, NF-κB is activated in response to intercellular signals such as cytokines, neurotransmitters, ion channels and neurotrophic factors (Gessi et al., 2016; Numata et al., 2016; Ren et al., 2016; Schlicher et al., 2016; Wang et al., 2016). It has been reported that NaHS can protect against water-immersion and restraint stress-induced gastric damage in rats via NF-κB-dependent pathway (Sun et al., 2017). Several studies have shown that H_2_S is mediated by the downregulation of NF-κB (Chattopadhyay et al., 2012; Wei et al., 2015; Ma et al., 2017). However, in this study, we found that NaHS inhibited gastric motility in rats, possibly through NF-κB-dependent signaling pathway. The differences between our results and prior findings might be attributed to the different cellular and animal models used.

In conclusion, our findings indicate that exogenous H_2_S within the NA significantly inhibits gastrointestinal motility in rats. It may occur through the activation of TRPV1 channels and NF-κB-dependent mechanism. This is the first study to elucidate the role of H_2_S as a promising regulator of gastrointestinal motility in the NA.

## Data Availability Statement

All datasets presented in this study are included in the article/supplementary material.

## Ethics Statement

Ethical approval for this study was obtained from the Experimental Animal Ethics Committee of Qilu Normal University.

## Author Contributions

HoS and HaS conceived and designed the experiments. HD, YS, CL, HJ, XY, ZC, and PT performed the experiments. HoS and HD analyzed the data. HoS and JZ contributed reagents, materials, and analysis tools. HoS wrote the manuscript. HoS and HaS participated in the redaction and correction of the manuscript. All authors contributed to the article and approved the submitted version.

## Conflict of Interest

The authors declare that the research was conducted in the absence of any commercial or financial relationships that could be construed as a potential conflict of interest.
